# Supplementary material for: Single cell transcriptomics clarifies the basophil differentiation trajectory and identifies pre-basophils upstream of mature basophils
Source: Nat Commun. 2023 May 18;14:2694. doi: 10.1038/s41467-023-38356-1 (PMC10195816; doi:10.1038/s41467-023-38356-1)
Supplement: Supplementary file 1 — Supplementary Information [file 41467_2023_38356_MOESM1_ESM.pdf]

Supplementary Figures:

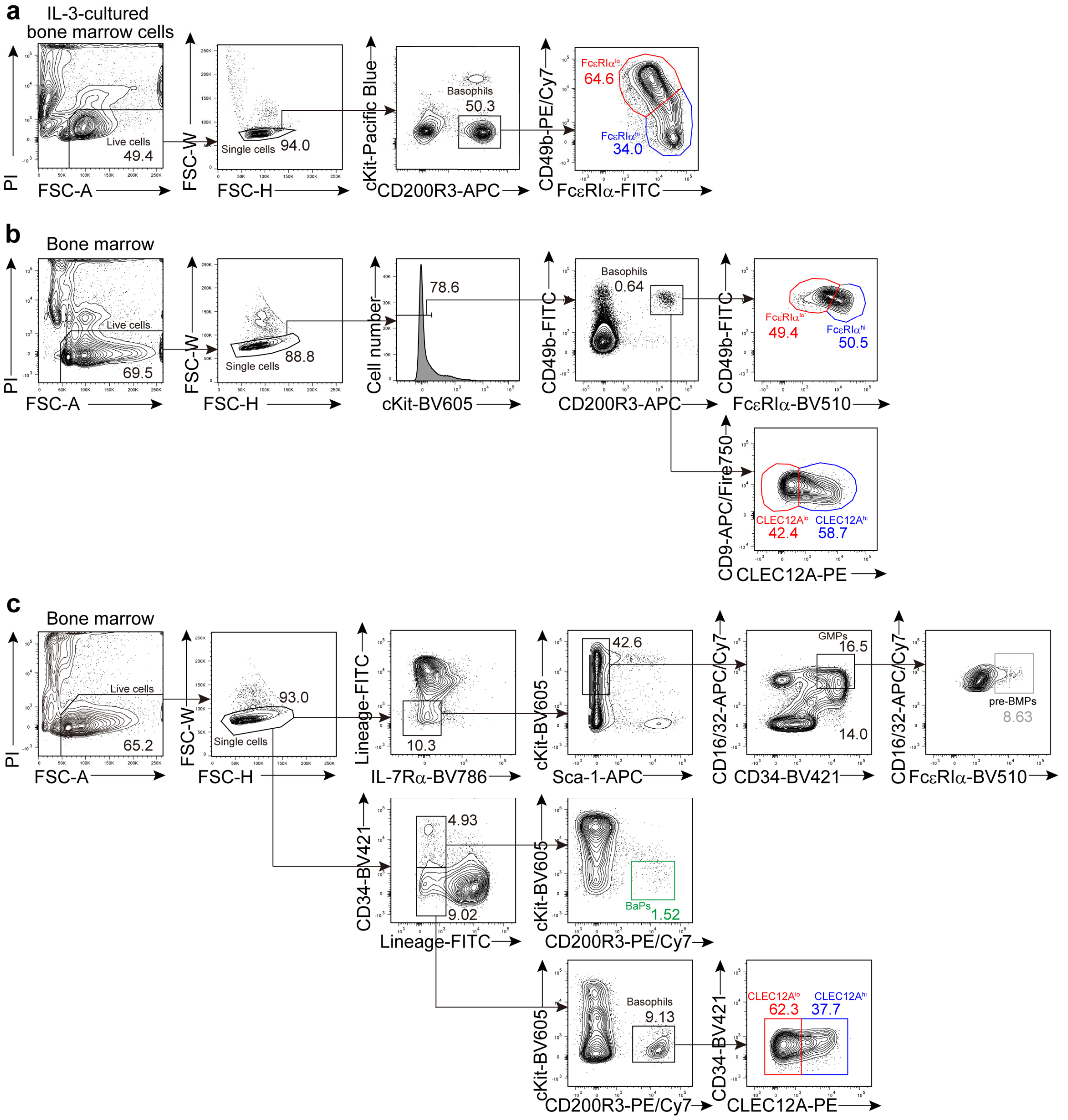

Supplementary Fig. 1. **Flow cytometry gating strategy for BMBAs and bone marrow basophils.** (a) Gating strategy in flow cytometric analysis to identify basophils among the IL-3-cultured bone marrow cells is shown. (b) Gating strategy in flow cytometric analysis to identify basophils in the bone marrow is shown. (c) Gating strategy in flow cytometric analysis to identify pre-BMPs, CD34^{+} BaPs and CD34^{-} basophils in the bone marrow is shown.

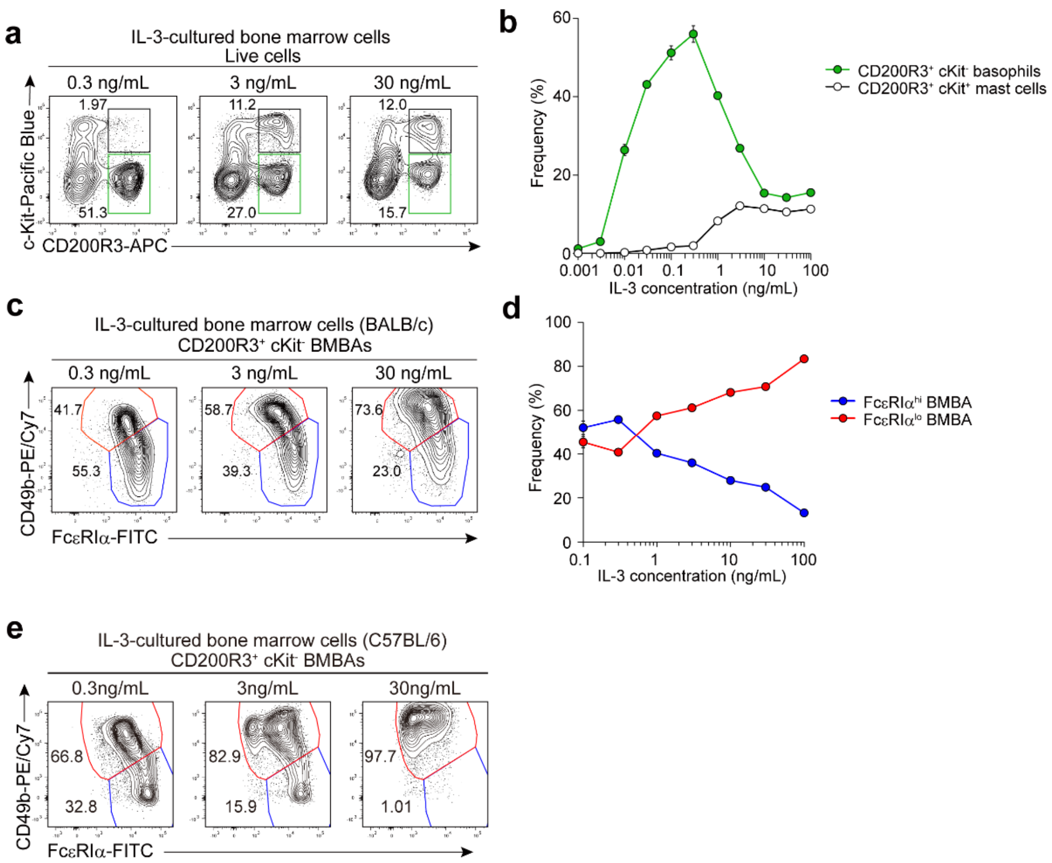

Supplementary Fig. 2. BMBAs consist of two distinct subpopulations. (a-d) Bone marrow cells of BALB/c mice were cultured in the presence of varying concentrations of IL-3 for 7 days. CD200R3^{+}cKit^{-} basophil (BMBA) fraction and CD200R3^{+}cKit^{+} mast cell fraction in the culture were separately gated (a), and the frequency of each fraction among total cells cultured with indicated concentration of IL-3 is shown (b, mean ± SEM, n=3 each). CD200R3^{+}cKit^{-} BMBAs were further divided into FcεRIα^{hi}CD49b^{lo} and FcεRIα^{lo}CD49b^{hi} subpopulations (c), and the frequency of each subpopulation among BMBAs generated in the presence of indicated concentration of IL-3 is shown (d, mean ± SEM, n=3 each). (e) Bone marrow cells of C57BL/6 mice were cultured in the presence of indicated concentrations of IL-3 for 7 days. The surface expression of FcεRIα and CD49b is shown.

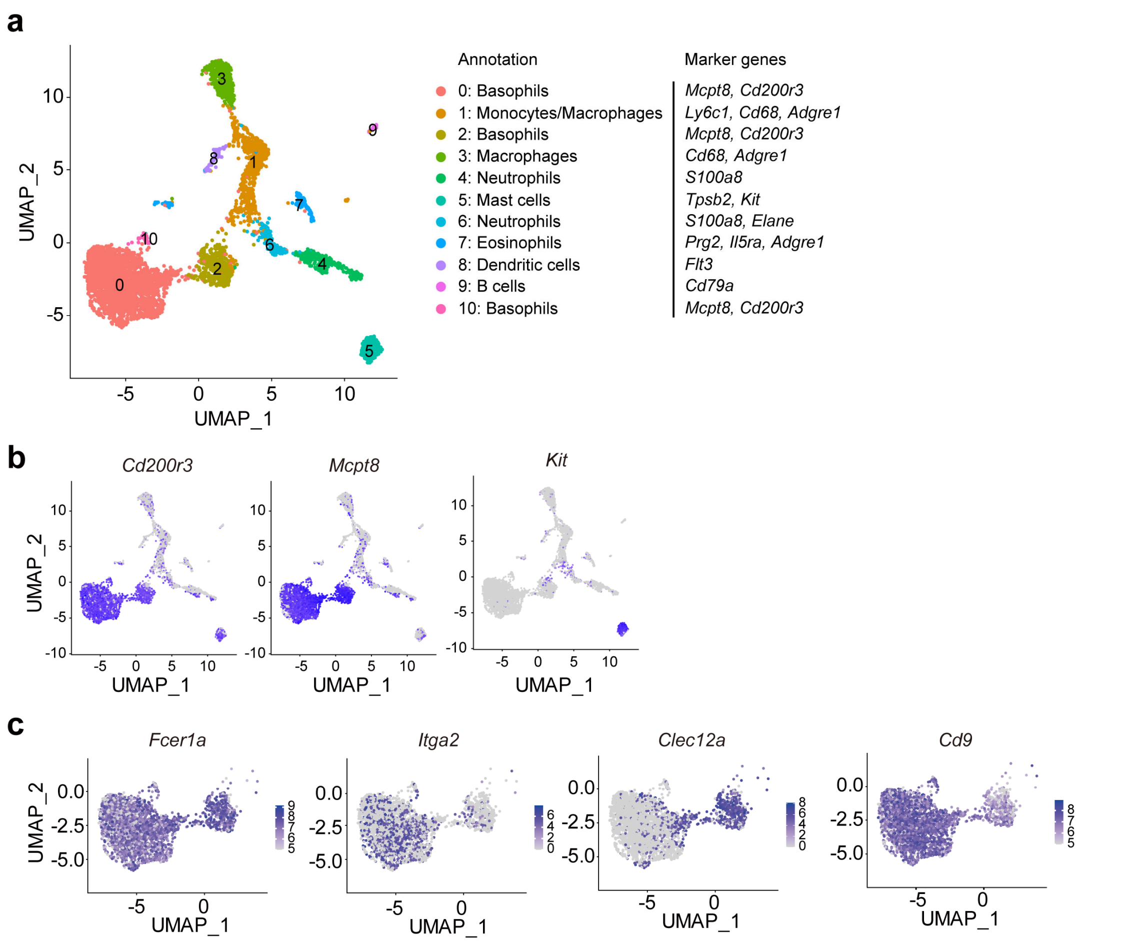

Supplementary Fig. 3. Single-cell RNA-seq analysis identifies heterogeneous subpopulations among BMBAs. Bone marrow cells were cultured in the presence of 0.3 ng/ml of IL-3 for 7 days and subjected to single-cell RNA-seq (scRNA-seq) analysis. (a) UMAP plot of total cells is shown. (b) Feature plots showing the expression of indicated genes. (c) Feature plots of basophil clusters (clusters 0, 2, and 10 depicted in a) showing the expression of *Fcer1a, Itga2, Clec12a* and *Cd9*.

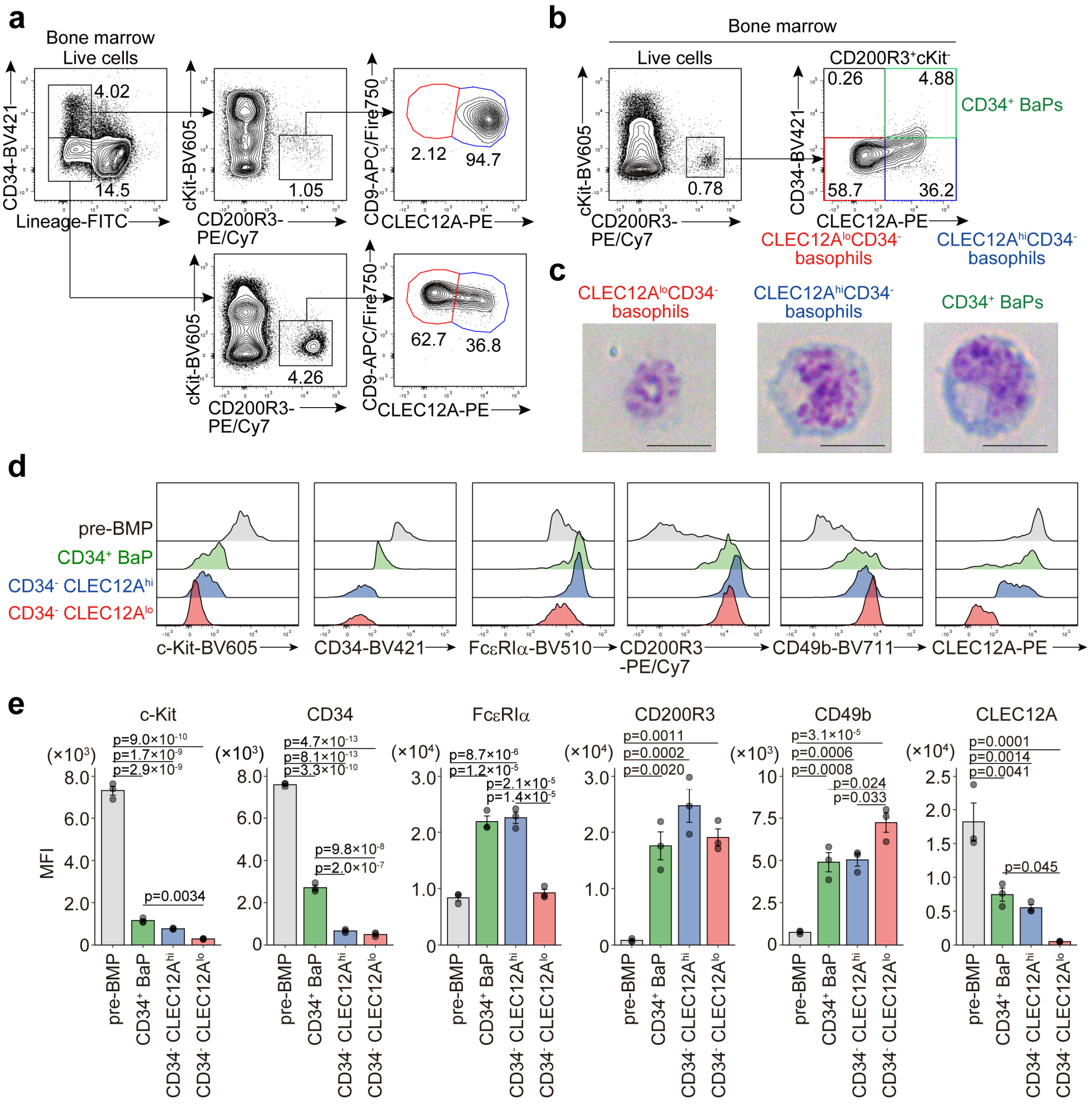

Supplementary Fig. 4. CD34$^+$ BaPs display morphology and surface expression profiles similar to those of CLEC12A^{hi} basophils. (a) Lineage$^-$CD34$^+$cKit$^-$CD200R3$^+$ basophil precursors (BaPs) and Lineage$^-$CD34$^-$cKit$^-$CD200R3$^+$ basophils in the bone marrow were gated (left and middle panels), and surface expression of CLEC12A and CD9 (right panels) is shown. (b) CD45$^+$cKit$^-$CD200R3$^+$ basophils in the bone marrow were gated (left panel) and surface expression of CLEC12A and CD34 (right panel) is shown. (c) CLEC12A^{lo}CD34$^-$, CLEC12A^{hi}CD34$^-$, CLEC12A^{hi}CD34$^+$ subpopulations of basophils in the bone marrow were separately sort-purified and stained with May-Grünwald Giemsa (scale bar, 10 μm). (d and e) The surface expression (d) and MFI (e) of indicated molecules in pre-BMPs, CD34$^+$ BaPs, CD34$^-$CLEC12A^{hi} basophils, and CD34$^-$CLEC12A^{lo} basophils are shown (mean ± SEM, n=3 each). Data are representative of two independent experiments. One-way ANOVA with Tukey's multiple comparisons test was used for multiple comparisons (e).

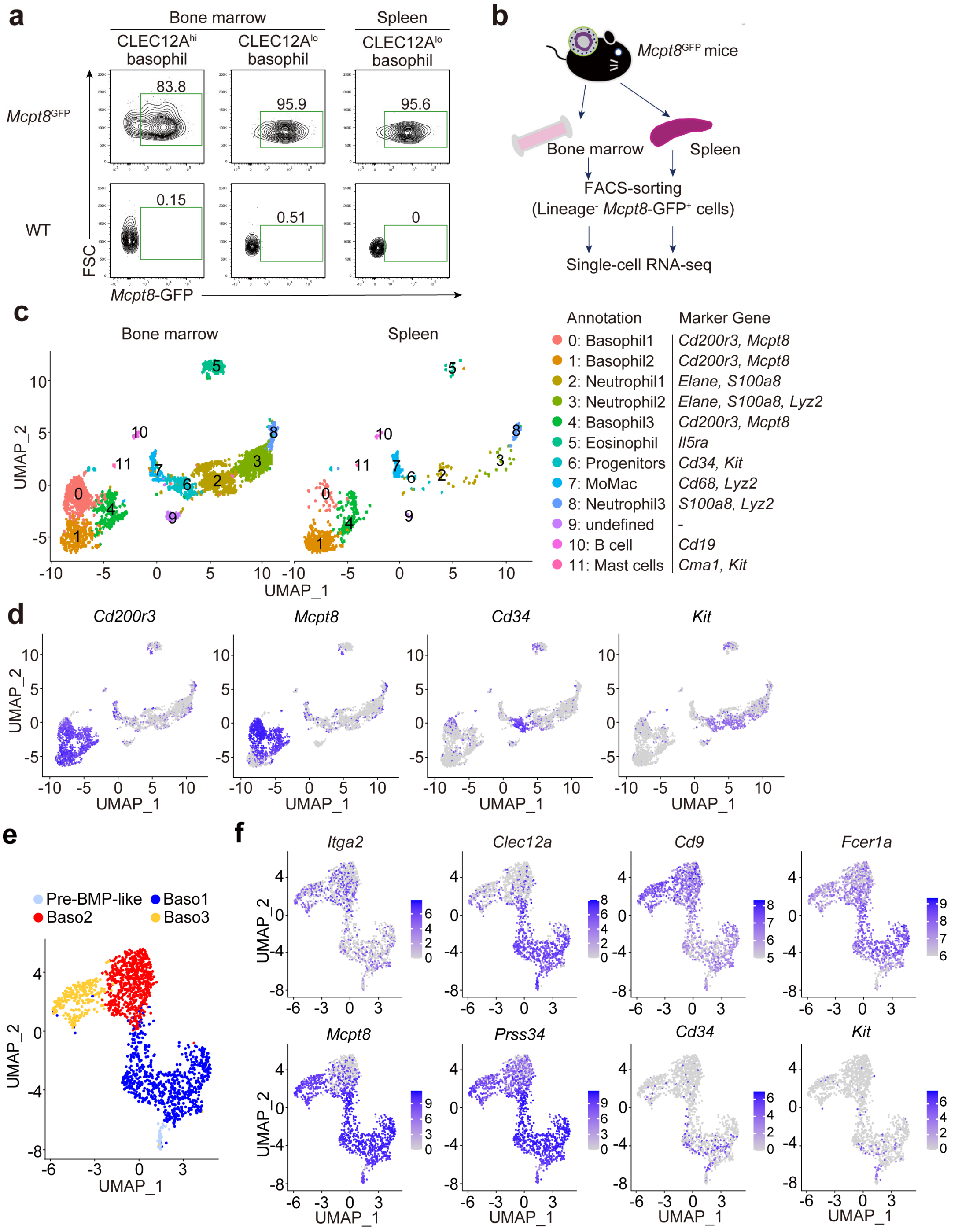

Supplementary Fig. 5. Single-cell RNA-seq analysis identifies different clusters among bone marrow basophils. (a) GFP expression in CLEC12A^{hi} and CLEC12A^{lo} bone marrow basophils and CLEC12A^{lo} spleen basophils isolated from *Mcpt8*^{GFP} mice. (b-e) Lineage^{-}GFP^{+} bone marrow and spleen cells were isolated from *Mcpt8*^{GFP} mice and separately subjected to scRNA-seq analysis. (c) UMAP plots of GFP^{+} bone marrow cells (left panel) and GFP^{+} splenocytes (right panel) are shown. (d) Feature plots showing the expression of indicated genes in combined dataset of the bone marrow and spleen. (e)

UMAP plot of combined data set of 2,745 cells from bone marrow basophils and 731 cells from spleen basophils is shown. (f) Basophil clusters (clusters 0, 1, and 4) identified in c were re-clustered for further analysis. Feature plots of basophil populations displaying the expression of indicated genes are shown.

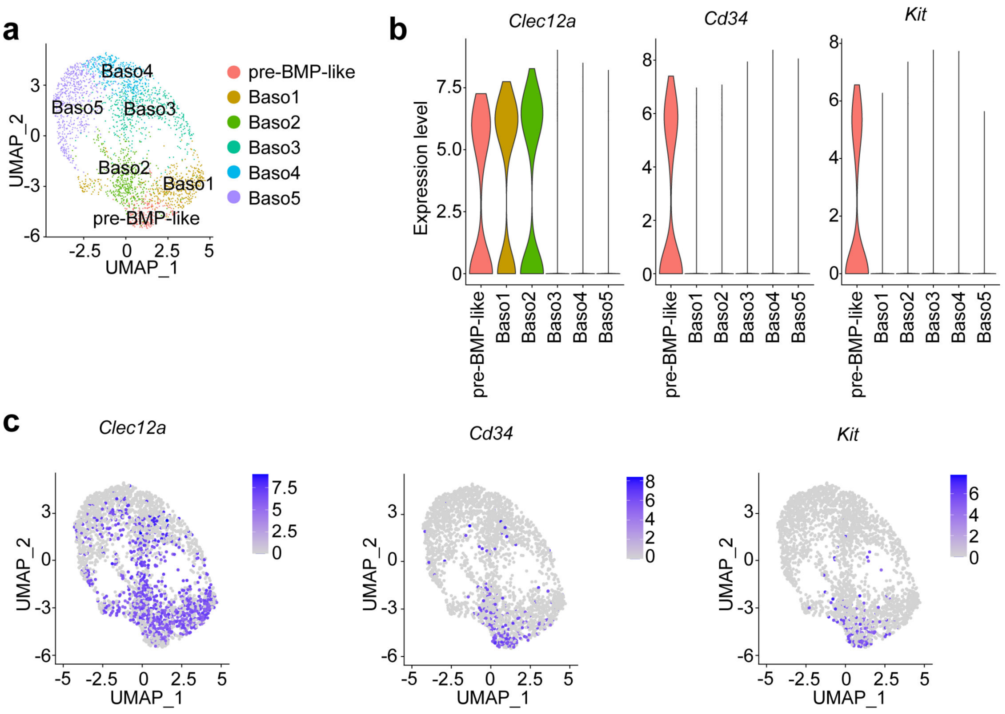

Supplementary Fig. 6. **Re-analysis of basophil populations in GSE140802.** The *Cd200r3*$^{+}$*Mcpt8*$^{+}$ basophil population in GSE140802 was re-clustered. (a) UMAP plot of the basophil population. Colors indicate different Seurat clusters. (b) Violin plots of the expression of indicated genes in each cluster. (c) Feature plots displaying the expression of indicated genes. The frequency of *Cd34*$^{+}$ cells in each cluster is described in this plot (middle panel).

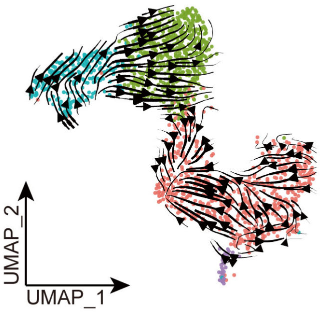

Supplementary Fig. 7. **RNA velocity analysis identifies the differentiation trajectory of basophils.** RNA velocity analysis was conducted on the scRNA-seq dataset presented in Fig. 2e. RNA velocities derived from dynamical model are visualized as streamlines in a UMAP-based embedding.

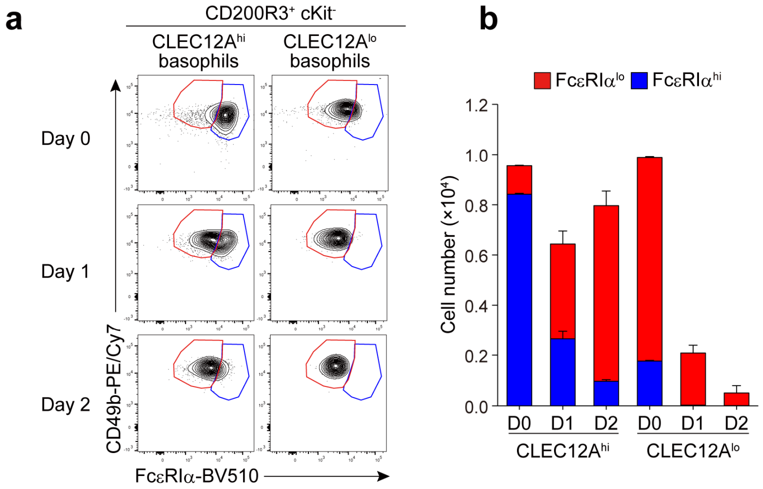

Supplementary Fig. 8. **FcεRIα^{hi}CD49b^{lo} basophils differentiate to FcεRIα^{lo}CD49b^{hi} basophils.** Sort-purified CLEC12A^{hi}CD9^{lo} and CLEC12A^{lo}CD9^{hi} bone marrow basophils were separately cultured *ex vivo* for 1 or 2 days. (a) Time course of FcεRIα and CD49b expression in the culture without IL-3 is shown. (b) The change in the number of live cells during the culture is shown; the red and blue bars correspond to the FcεRIα^{lo}CD49b^{hi} and FcεRIα^{hi}CD49b^{lo} fractions, respectively (mean ± SEM, n=3 each). (c) The separated graphs for each experiment in Figure 3c is shown (mean value is shown).

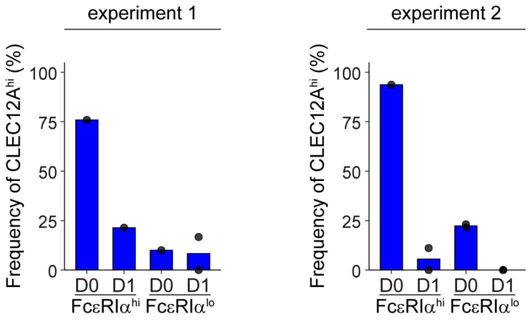

Supplementary Fig. 9. CLEC12A^{hi}CD9^{lo} basophils differentiate into CLEC12A^{lo}CD9^{hi} basophils *in vivo*. FcεRIα^{hi}CD49b^{lo} and FcεRIα^{lo}CD49b^{hi} subpopulations of basophils were separately isolated from the bone marrow of CD45.2^{+} C57BL/6 mice (5 × 10^{4} cells/mouse), mixed with CD45.1^{+} whole bone marrow cells (0.3 × 10^{6} cells/mouse), and intravenously administered to sublethally-irradiated CD45.1^{+} congenic C57BL/6 mice as in Fig. 3d. The frequency of CLEC12A^{hi} cells before and 1 day after the transfer in each experimental group is shown where data are displayed for two separated experiments (mean ± SEM, for experiment 1, n=1 for FcεRIα^{hi} on D0, n=1 for FcεRIα^{lo} on D0, n=1 for FcεRIα^{hi} on D1, n=2 for FcεRIα^{lo} on D1; for experiment 2, n=1 for FcεRIα^{hi} on D0, n=2 for FcεRIα^{lo} on D0, n=2 for FcεRIα^{hi} on D1, n=3 for FcεRIα^{lo} on D1).

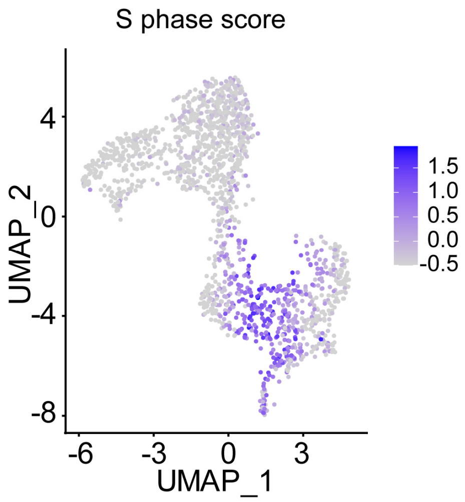

Supplementary Fig. 10. **S phase scores shown in the feature plot of the scRNA-seq data of basophil clusters.** Feature plots of scRNA-seq data of basophil clusters (Fig. 2e) displaying the S phase score.

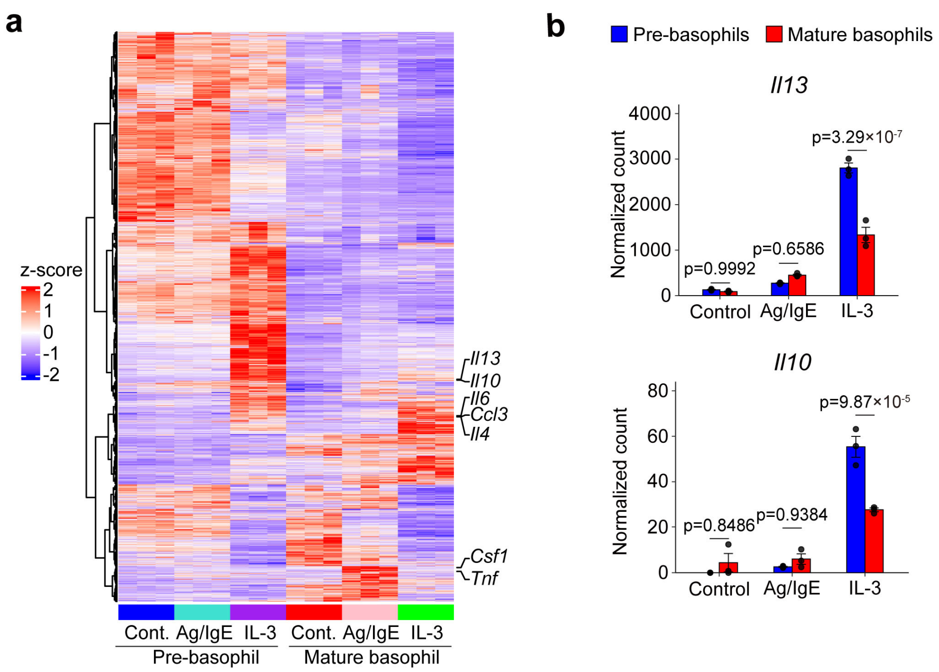

Supplementary Fig. 11. **IL-3-stimulated pre-basophils display gene expression profiles distinct from those of activated mature basophils.** Mice were intravenously sensitized with anti-TNP IgE 1 day prior to the experiment. CLEC12A^{hi} and CLEC12A^{lo} basophils isolated from the bone marrow of TNP-IgE-sensitized mice were separately cultured *ex vivo* in the presence of OVA (Cont.), TNP-OVA [antigen (Ag)/IgE) or IL-3 for 4 h and subjected to bulk RNA-seq analysis (n=3 cultures, each). (a) Hierarchically clustered heatmap of differentially expressed genes is shown. (b) Normalized counts of the indicated genes are shown (mean ± SEM, n = 3 each). Two-way ANOVA with Tukey's multiple comparisons test was used for multiple comparisons (b).

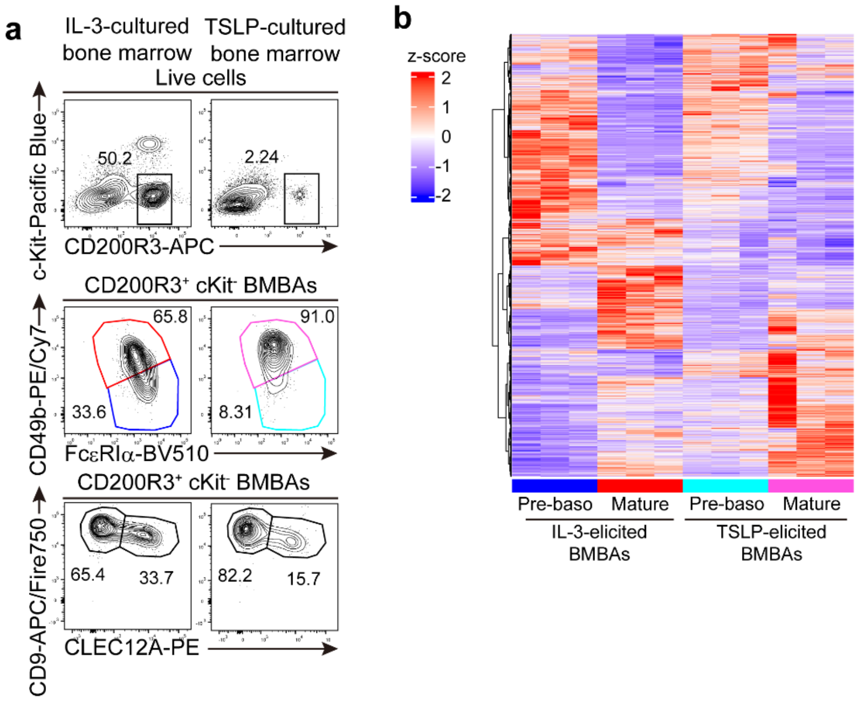

Supplementary Fig. 12. **Gene expression profiles of IL-3- or TSLP-elicited BMBAs.** Bone marrow cells were cultured with IL-3 (0.3 ng/mL) or TSLP (1 μg/mL) for 7 days. (a) CD200R3^{+}cKit^{-} BMBAs were gated (upper panels) and their surface expression of FcεRIα and CD49b (middle panel) or CLEC12A and CD9 (bottom panel) is shown. (b) FcεRIα^{hi}CD49b^{lo} pre-basophils and FcεRIα^{lo}CD49b^{hi} mature basophils isolated from either IL-3- or TSLP-elicited BMBAs were separately subjected to bulk RNA-seq analysis (n=3 cultures, each). The hierarchically clustered heatmap of DEGs in pre-basophils and mature basophils isolated from either IL-3-elicited or TSLP-elicited BMBAs is shown.

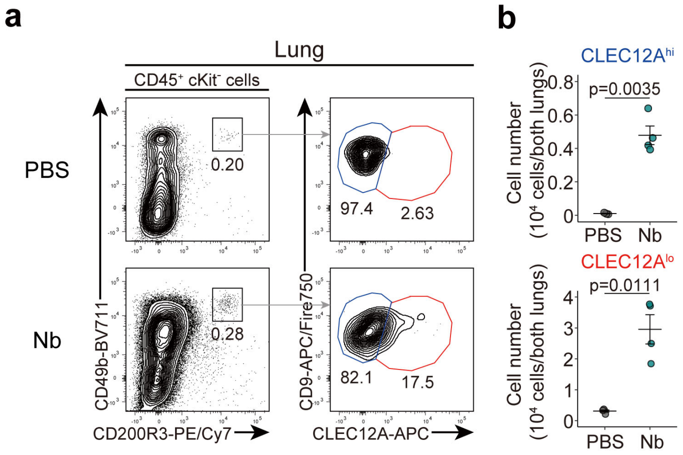

Supplementary Fig. 13. **Nb infection but not PBS treatment promotes the emergence of pre-basophils in the lungs.** C57BL/6 mice were infected with Nb larvae or treated with intradermal injection of PBS as control, and cells were prepared from the lungs on day 7 post-infection. The expression of CLEC12A and CD9 on $cKit^{-}CD200R3^{+}CD49b^{+}$ basophils (a) and cell numbers of the $CLEC12A^{hi}$ and $CLEC12A^{lo}$ basophils (b) are shown (mean ± SEM, n=3 each). Data are representative of three independent experiments. Unpaired Student's *t*-test was used for comparing two groups (b).

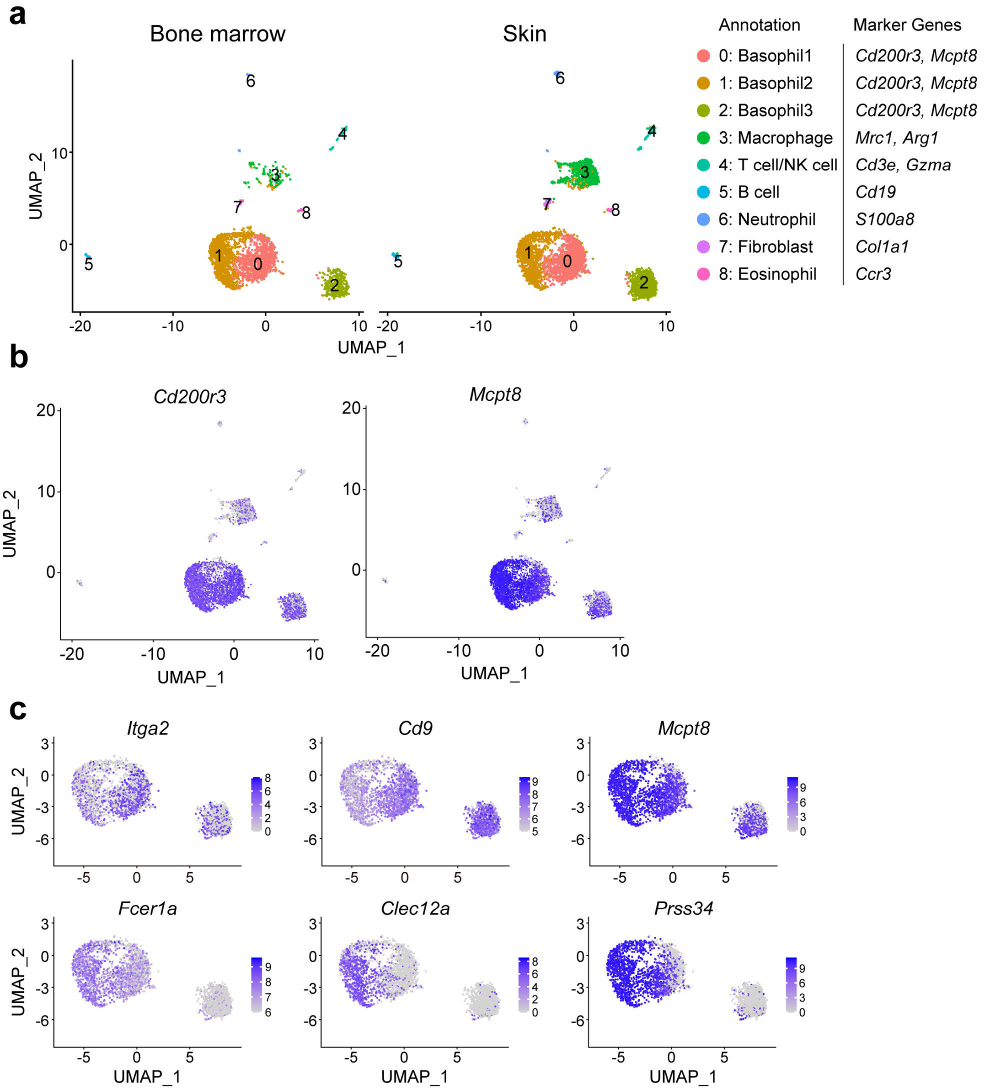

Supplementary Fig. 14. **scRNA-seq analysis of cells in the bone marrow and the skin lesion of mice infected twice with Nb.** C57BL/6 mice were infected twice with Nb larvae. CD200R3^{+}cKit^{-} cells were isolated from the bone marrow and infected skin and subjected to scRNA-seq analysis. (a) UMAP plots of bone marrow cells (left panel) and skin cells (right panel) are shown. (b) Feature plots displaying the expression of indicated genes are shown. (c) Feature plots of basophil clusters (clusters 0,1, and 2 depicted in a) showing the expression of indicated genes.

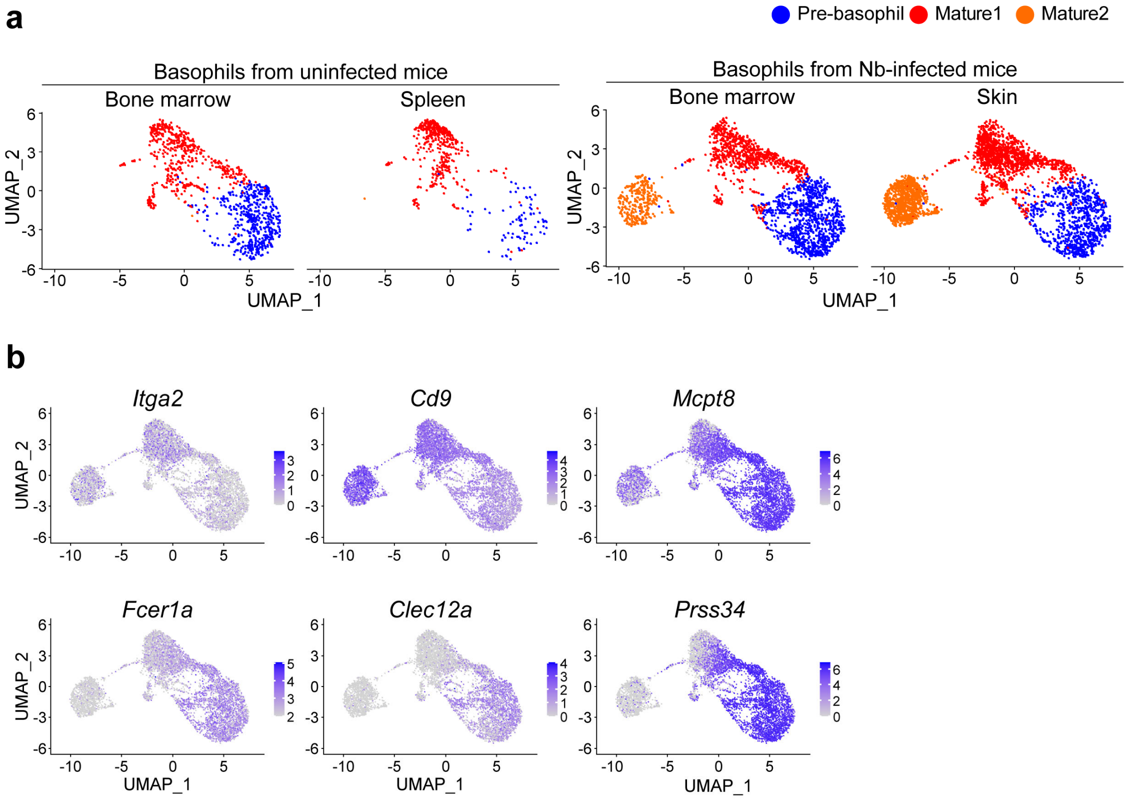

Supplementary Fig. 15. **Integration of scRNA-seq analysis reveals the presence of pre-basophils in the Nb-infected skin.** Basophil clusters shown in Fig. 2e and Fig. 5e were integrated for further analysis. (**a**) UMAP plots of bone marrow and spleen basophils in uninfected mice are shown in the left panels. UMAP plots of bone marrow and skin basophils in mice infected twice with Nb are shown in the right panels. (**b**) Feature plots showing the expression of indicated genes are shown.

a

b

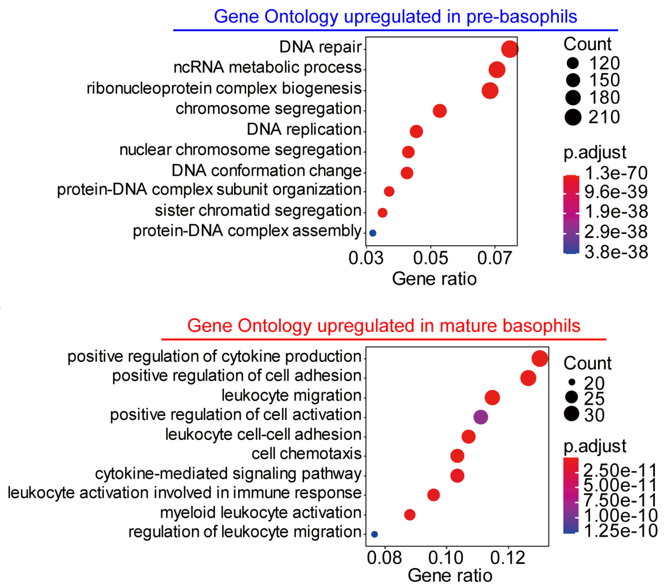

Supplementary Fig. 16. Pre-basophils in the Nb-infected skin show higher expression of cell proliferation-related genes compared to mature basophils. The top 10 enriched GO terms upregulated in the pre-basophil cluster (a) and in the mature basophil clusters (b) in the Nb-infected skin (Fig. 5e), respectively, are plotted in order of gene ratio. The size of the dots indicates the number of genes associated with indicated GO terms (Count) while the color of the dots indicates the adjusted *P*-values (p. adjust) calculated by one-sided Fisher's exact test with Benjamini-Hochberg correction.

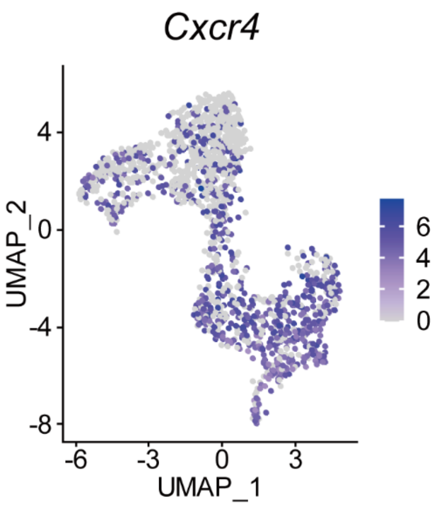

Supplementary Fig. 17. ***Cxcr4* expression of basophils in the scRNA-seq data.** The expression of *Cxcr4* is shown in the feature plot of the scRNA-seq data of basophil clusters (Fig. 2e)
