# Supplementary material for: Single cell transcriptomics clarifies the basophil differentiation trajectory and identifies pre-basophils upstream of mature basophils
Source: Nat Commun. 2023 May 18;14:2694. doi: 10.1038/s41467-023-38356-1 (PMC10195816; doi:10.1038/s41467-023-38356-1)
Supplement: Supplementary file 3 — Reporting Summary [file 41467_2023_38356_MOESM3_ESM.pdf]

nature portfolio

Corresponding author(s): Kensuke Miyake

Last updated by author(s): Mar 31, 2023

Reporting Summary

Nature Portfolio wishes to improve the reproducibility of the work that we publish. This form provides structure for consistency and transparency in reporting. For further information on Nature Portfolio policies, see our Editorial Policies and the Editorial Policy Checklist.

Statistics

For all statistical analyses, confirm that the following items are present in the figure legend, table legend, main text, or Methods section.

n/a	Confirmed	
☐	☒	The exact sample size (*n*) for each experimental group/condition, given as a discrete number and unit of measurement
☐	☒	A statement on whether measurements were taken from distinct samples or whether the same sample was measured repeatedly
☐	☒	The statistical test(s) used AND whether they are one- or two-sided<br>*Only common tests should be described solely by name; describe more complex techniques in the Methods section.*
☒	☐	A description of all covariates tested
☐	☒	A description of any assumptions or corrections, such as tests of normality and adjustment for multiple comparisons
☐	☒	A full description of the statistical parameters including central tendency (e.g. means) or other basic estimates (e.g. regression coefficient) AND variation (e.g. standard deviation) or associated estimates of uncertainty (e.g. confidence intervals)
☐	☒	For null hypothesis testing, the test statistic (e.g. *F*, *t*, *r*) with confidence intervals, effect sizes, degrees of freedom and *P* value noted<br>*Give P values as exact values whenever suitable.*
☒	☐	For Bayesian analysis, information on the choice of priors and Markov chain Monte Carlo settings
☐	☒	For hierarchical and complex designs, identification of the appropriate level for tests and full reporting of outcomes
☒	☐	Estimates of effect sizes (e.g. Cohen's *d*, Pearson's *r*), indicating how they were calculated

Our web collection on statistics for biologists contains articles on many of the points above.

Software and code

Policy information about availability of computer code

Data collection	For bulk and single-cell RNA-seq analysis, cDNA libraries were sequenced by using Novaseq 6000 (Illumina) or Ion Torrent Proton (Thermo Fisher) as described in Methods section. No commercial, open source or custom code was used to collect data.
Data analysis	For bulk RNA-seq data, single-end Fastq files were processed as follows: Adapter trimming of sequencing data was performed by using cutadapt 3.4, and trimmed reads were mapped to reference RNA (build GRCm38 release-101) by using bowtie2-2.4.2 by the following parameters: -p 8 -N 1 -L 16 –very-sensitive-local –seed 656565 –nofw. Then, read counts of each gene in each sample were counted by using awk, sort, and uniq -c commands. Resulted gene-expression count data was summarized by same Gene Symbol and all of the expression table was full-outer joined by Gene Symbol by using dplyr-1.0.7 package. Normalization of count data and the detection of differential expressed genes were performed by using TCC v.1.32.0. GO enrichment analysis was performed by the R software package clusterProfiler v4.0.5.<br><br>For scRNA-seq analysis, pair-end Fastq data were processed as follows: Adapter trimming of sequencing data was performed by using cutadapt 2.10. Filtered reads were chunked to 16 parts for parallel processing by using Seqkit 0.9.0. Filtered cell barcode reads were annotated by Python script provided by BD with minor modification for compatible to Python3.7. Associated cDNA reads were mapped to reference RNA (build GRCm38 release-101) by using bowtie2-2.4.2 by the following parameters: -p 2 -D 20 -R 3 -N 0 -L 8 -i S,1,0.75 –norc –seed 656565 –reorder. Then, cell barcode information of each read was added to the bowtie2-mapped BAM files, and read counts of each gene in each cell barcode were counted by using mawk. Resulted count data was converted to genes x cells matrix file and inflection threshold of the knee-plot was detected by using DropletUtils package in R 3.6.3. In addition, we further estimate background beads by using emptyDrops formula in DropletUtils package.<br>The resultant dataset was analyzed using R software package Seurat v4.0.4 in R 4.1.0. After the removal of doublets from the dataset, the log-normalized gene counts were calculated using NormalizeData function (scale.factor = 1,000,000) and highly variable genes were defined by FindVariableFeatures function (selection.method=”vst”, nfeature=2000). To mitigate the effect of cell cycle heterogeneity and the difference

in read counts, cell cycle scores were calculated using CellCycleScoring function. Read counts and the difference between the G2M and S phase scores were regressed out by the ScaleData function. Principal component analysis was performed on the variable genes, and principal components with their p-value <0.05 calculated by the jackstraw method, were subjected to cell clustering (resolution=0.2 for Fig.1 and 5: 1.0 for Fig.2) and UMAP dimensional reduction. For Figure 2, basophil-lineage clusters were subjected to re-clustering analysis. For the joint analysis of basophils in steady state mice (Fig.2) and Nb-infected mice (Fig.5), basophil clusters in these two scRNA-seq datasets were merged by the SelectIntegrationFeatures function, FindIntegrationAnchors function, and IntegrateData function. Differentially expressed genes were defined as those whose p-value, as calculated by the Wilcoxon rank sum test and adjusted by the Bonferroni method is <0.05 and whose log2FoldChange is >0.5 or <-0.5. GSEA and GO enrichment analysis were conducted by utilizing the R software package clusterProfiler v4.0.5 . Pseudotime analysis was performed by utilizing monocle3 v1.0.1. RNA velocity analysis was conducted by utilizing scVelo.
The R codes for bulk and single-cell RNA-seq analysis are available at GitHub (https://github.com/KensukeMiyake/Pre-basophil-paper).

For flow cytometric analysis, FlowJo ver 10.8.1 (BD Biosciences) was used to analyze FACS data.

For statistical analysis, GraphPad Prism (ver 7.0.3) or R software (ver 4.1.0) are used.

For manuscripts utilizing custom algorithms or software that are central to the research but not yet described in published literature, software must be made available to editors and reviewers. We strongly encourage code deposition in a community repository (e.g. GitHub). See the Nature Portfolio guidelines for submitting code & software for further information.

Data

Policy information about availability of data

All manuscripts must include a data availability statement. This statement should provide the following information, where applicable:
- Accession codes, unique identifiers, or web links for publicly available datasets
- A description of any restrictions on data availability
- For clinical datasets or third party data, please ensure that the statement adheres to our policy

Transcriptomic data generated in this study have been deposited in Gene Expression Omnibus (GEO) under accession numbers GSE206589 [https://www.ncbi.nlm.nih.gov/geo/query/acc.cgi?acc=GSE206589], GSE206590 [https://www.ncbi.nlm.nih.gov/geo/query/acc.cgi?acc=GSE206590], GSE206591 [https://www.ncbi.nlm.nih.gov/geo/query/acc.cgi?acc=GSE206591], GSE206592 [https://www.ncbi.nlm.nih.gov/geo/query/acc.cgi?acc=GSE206592], GSE206593 [https://www.ncbi.nlm.nih.gov/geo/query/acc.cgi?acc=GSE206593], and GSE206631 [https://www.ncbi.nlm.nih.gov/geo/query/acc.cgi?acc=GSE206631]. For mapping of transcriptomic data, mouse Ensembl RNA (GRCm38.p6, release-101; http://aug2020.archive.ensembl.org/Mus_musculus/Info/Index) was used.

Human research participants

Policy information about studies involving human research participants and Sex and Gender in Research.

Reporting on sex and gender	n/a
Population characteristics	n/a
Recruitment	n/a
Ethics oversight	n/a

Note that full information on the approval of the study protocol must also be provided in the manuscript.

Field-specific reporting

Please select the one below that is the best fit for your research. If you are not sure, read the appropriate sections before making your selection.

[x] Life sciences [ ] Behavioural & social sciences [ ] Ecological, evolutionary & environmental sciences

For a reference copy of the document with all sections, see nature.com/documents/nr-reporting-summary-flat.pdf

Life sciences study design

All studies must disclose on these points even when the disclosure is negative.

Sample size	No statistical methods were used to determine sample size. The sample size was more than 3 per group. According to the 3R principle, we chose the minimal replicate number sufficient to ascertain statistics by unpaired t-test, one-way ANOVA, or two-way ANOVA.
Data exclusions	No data were excluded from mice experiments.
Replication	All the experiments were successfully replicated at least two times (Most data were successfully replicated more than three times).
Randomization	Animals used in this study were randomly assigned to their respective groups before the experiments were performed.

Blinding | Blinding was not achieved in this study due to requirements for cage identification and labeling for treatment purposes.

Reporting for specific materials, systems and methods

We require information from authors about some types of materials, experimental systems and methods used in many studies. Here, indicate whether each material, system or method listed is relevant to your study. If you are not sure if a list item applies to your research, read the appropriate section before selecting a response.

Materials & experimental systems

n/a	Involved in the study
☐	☒ Antibodies
☒	☐ Eukaryotic cell lines
☒	☐ Palaeontology and archaeology
☐	☒ Animals and other organisms
☒	☐ Clinical data
☒	☐ Dual use research of concern

Methods

n/a	Involved in the study
☒	☐ ChIP-seq
☐	☒ Flow cytometry
☒	☐ MRI-based neuroimaging

Antibodies

Antibodies used

The following antibodies were purchased from BioLegend:
Biotin anti-mouse/human CD45R/B220 Antibody (clone: RA3-6B2, catalog #: 103204, Lot: B268525),
Biotin anti-mouse CD3ε Antibody (clone: 145-2C11, catalog #: 100304, Lot: B268248),
Biotin anti-mouse CD4 Antibody (clone: GK1.5, catalog #: 100404, Lot: B191781),
Biotin anti-mouse CD8a Antibody (clone: 53-6.7, catalog #: 100704, Lot: B215537),
Biotin anti-mouse CD19 Antibody (clone: 6D5, catalog #: 115504, Lot: B226661),
Biotin anti-mouse CD49b (pan-NK cells) Antibody (clone: DX5, catalog #: 108904, Lot: B285502),
Biotin anti-mouse Ly-6G/Ly-6C (Gr-1) Antibody (clone: RB6-8C5, catalog #: 108404, Lot: B236916),
Biotin anti-mouse TER-119/Erythroid Cells Antibody (clone: TER-119, catalog #: 116204, Lot: B193723);
FITC anti-mouse CD49b Antibody (clone: HMα2, catalog #: 103504, Lot: B250460),
FITC anti-mouse FcεRIα Antibody (clone: MAR-1, catalog #: 134306, Lot: B151021),
FITC anti-mouse CD45 Antibody (clone: 30-F11, catalog #: 103108, Lot: B330230);
Alexa Fluor 488 anti-mouse CD45.2 Antibody (clone: 104, catalog #: 109816, Lot: B234286);
PE anti-mouse CD34 Antibody (clone: SA376A4, catalog #: 152204, Lot: B311897),
PE anti-mouse CD63 Antibody (clone: NVG-2, catalog #: 143904, Lot: B245564),
PE anti-mouse CD371 (CLEC12A) Antibody (clone: 5D3/CLEC12A, catalog #: 143404, Lot: B309771),
PE anti-mouse CD184 (CXCR4) Antibody (clone: L276F12, catalog #: 146506, Lot: B337979),
PE Rat IgG2a, κ Isotype Ctrl Antibody (clone: RTK2758, catalog #: 400508, Lot: B316163)
PE Rat IgG2b, κ Isotype Ctrl Antibody (clone: RTK4530, catalog #: 400608, Lot: B156144);
PE/Cyanine7 anti-mouse CD49b Antibody (clone: HMa2, catalog #: 103518, Lot: B348493)
PE/Cyanine7 anti-mouse CD200R3 Antibody (clone: Ba13, catalog #: 142212, Lot: B297486);
APC anti-mouse CD200R3 Antibody (clone: Ba13, catalog #: 142208, Lot: B324365),
APC anti-mouse Ly-6A/E (Sca-1) Antibody (clone: D7, catalog #: 108112, Lot: B171677)
APC anti-mouse CD371 (CLEC12A) Antibody (clone: 5D3/CLEC12A, catalog #: 143406, Lot: B324774);
APC/Cyanine7 anti-mouse CD16/32 Antibody (clone: S17011E, catalog #: 156612, Lot: B321726);
APC/Fire 750 anti-mouse CD9 Antibody (clone: MZ3, catalog #: 124814, Lot: B341290);
Pacific Blue anti-mouse CD117 (c-Kit) Antibody (clone: 2B8, catalog #: 105820, Lot: B314152)
Pacific Blue anti-mouse CD45.1 Antibody (clone: A20, catalog #:110722, Lot: B191340);
Brilliant Violet 421 anti-mouse CD34 Antibody (clone: SA376A4, catalog #: 152208, Lot: B332607)
Brilliant Violet 421 anti-mouse CD45 Antibody (clone: 30-F11, catalog #: 103134, Lot: B314484);
Brilliant Violet 510 anti-mouse FcεRIα Antibody (clone: MAR-1, catalog #: 134327, Lot: B358821);
Brilliant Violet 605 anti-mouse CD117 (c-Kit) Antibody (clone: 2B8, catalog #: 105847, Lot: B362403);
Brilliant Violet 711 anti-mouse CD184 (CXCR4) Antibody (clone: L276F12, catalog #: 146517, Lot: B339304);
Brilliant Violet 785 anti-mouse CD127 (IL-7Rα) Antibody(clone: A7R34, catalog #: 135037, Lot: B325967);
PerCP/Cyanine5.5 anti-mouse TER-119/Erythroid Cells Antibody (clone: TER-119, catalog #: 116228, Lot: B356105).
TruStain FcX PLUS (anti-mouse CD16/32) Antibody (clone: S17011E, catalog#: 156604, Lot: B362118, dilution 1:200)
BD OptiBuild BV711-conjugated anti-CD49b (clone: HMa2, catalog #: 740704, Lot: 2018203) was purchased from BD Biosciences.
FITC-conjugated Streptavidin (catalog #: 11-4317-87, Lot: E00564-1632) was purchased from Thermo Fisher Scientific.
Antibody dilution was 1:400 for the flow cytometric antibody shown above, unless otherwise specified.
The following BioLegend antibody was used in vivo study.
LEAF Purified anti-mouse IL-3 Antibody (clone: MP2-8F8, catalog#: 503906, Lot: B207844)
The following antibodies for scRNA-seq were purchased from BioLegend:
TotalSeq-A0301 anti-mouse Hashtag 1 Antibody (clone: M1/42; 30-F11; catalog #: 155801, Lot: B272593, dilution 1:400)
TotalSeq-A0302 anti-mouse Hashtag 2 Antibody (clone: M1/42; 30-F11; catalog #: 155803, Lot: B272594, dilution 1:400)

TotalSeq-A0306 anti-mouse Hashtag 6 Antibody (clone: M1/42; 30-F11; catalog #: 155811, Lot: B305123, dilution 1:400)
TotalSeq-A0312 anti-mouse Hashtag 12 Antibody (clone: M1/42; 30-F11; catalog #: 155823, Lot: B264843, dilution 1:400)
TotalSeq-A0313 anti-mouse Hashtag 13 Antibody (clone: M1/42; 30-F11; catalog #: 155825, Lot: B264845, dilution 1:400)
TNP-specific IgE antibody was prepared from hybridoma (IgE-Lb4, purchased from ADCC) in our laboratory.

Validation

Antibodies were used according to the manufacturer's instructions, based on their provided methods of validation.
The following antibodies were purchased from BioLegend:
1) Biotin anti-mouse/human CD45R/B220 Antibody (clone: RA3-6B2)
Verified Reactivity: Mouse, Human; Application:FC - Quality tested,IHC-F - Verified
Each lot of this antibody is quality control tested by immunofluorescent staining with flow cytometric analysis. For flow cytometric staining, the suggested use of this reagent is ≤ 0.25 µg per 106 cells in 100 µl volume. For immunohistochemistry, a concentration range of 5.0 - 10 µg/mL is suggested. It is recommended that the reagent be titrated for optimal performance for each application.
2) Biotin anti-mouse CD3ε Antibody (clone: 145-2C11),
Verified Reactivity: Mouse; Application: FC - Quality tested, IHC-F - Verified
Each lot of this antibody is quality control tested by immunofluorescent staining with flow cytometric analysis. For flow cytometric staining, the suggested use of this reagent is ≤ 0.25 µg per 106 cells in 100 µl. For immunohistochemistry, a concentration range of 5.0 - 10 µg/mL is suggested. It is recommended that the reagent be titrated for optimal performance for each application.
3) Biotin anti-mouse CD4 Antibody (clone: GK1.5),
Verified Reactivity: Mouse, Application: FC - Quality tested
Each lot of this antibody is quality control tested by immunofluorescent staining with flow cytometric analysis. For flow cytometric staining, the suggested use of this reagent is ≤ 0.25 µg per 106 cells in 100 µl volume. It is recommended that the reagent be titrated for optimal performance for each application.
4) Biotin anti-mouse CD8a Antibody (clone: 53-6.7),
Verified Reactivity: Mouse, Application: FC - Quality tested, IHC - Reported in the literature, not verified in house
Each lot of this antibody is quality control tested by immunofluorescent staining with flow cytometric analysis. For flow cytometric staining, the suggested use of this reagent is ≤ 0.25 µg per 106 cells in 100 µl volume. It is recommended that the reagent be titrated for optimal performance for each application.
5) Biotin anti-mouse CD19 Antibody (clone: 6D5),
Verified Reactivity: Mouse, Application: FC - Quality tested
Each lot of this antibody is quality control tested by immunofluorescent staining with flow cytometric analysis. For flow cytometric staining, the suggested use of this reagent is ≤1.0 µg per million cells in 100 µl volume. It is recommended that the reagent be titrated for optimal performance for each application.
6) Biotin anti-mouse CD49b (pan-NK cells) Antibody (clone: DX5),
Verified Reactivity: Mouse, Application: FC - Quality tested
Each lot of this antibody is quality control tested by immunofluorescent staining with flow cytometric analysis. For flow cytometric staining, the suggested use of this reagent is ≤ 0.25 µg per 106 cells in 100 µl volume. It is recommended that the reagent be titrated for optimal performance for each application.
7) Biotin anti-mouse Ly-6G/Ly-6C (Gr-1) Antibody (clone: RB6-8C5),
Verified Reactivity: Mouse, Application:FC - Quality tested, IP, IHC, WB - Reported in the literature, not verified in house
Each lot of this antibody is quality control tested by immunofluorescent staining with flow cytometric analysis. The suggested use of this reagent is ≤ 0.25 µg per 106 cells in 100 µl volume. It is recommended that the reagent be titrated for optimal performance for each application.
8) Biotin anti-mouse TER-119/Erythroid Cells Antibody (clone: TER-119)
Verified Reactivity: Mouse, Application: FC - Quality tested
Each lot of this antibody is quality control tested by immunofluorescent staining with flow cytometric analysis. For flow cytometric staining, the suggested use of this reagent is ≤ 0.25 µg per 106 cells in 100 µl volume. It is recommended that the reagent be titrated for optimal performance for each application.
9) FITC anti-mouse CD49b Antibody (clone: HMα2, catalog #: 103504)
Verified Reactivity: Mouse, Application: FC - Quality tested
Each lot of this antibody is quality control tested by immunofluorescent staining with flow cytometric analysis. For flow cytometric staining, the suggested use of this reagent is ≤ 0.25 µg per 106 cells in 100 µl volume. It is recommended that the reagent be titrated for optimal performance for each application.
10) FITC anti-mouse FcεRIα Antibody (clone: MAR-1),
Verified Reactivity: Mouse, Application: FC - Quality tested
Each lot of this antibody is quality control tested by immunofluorescent staining with flow cytometric analysis. For flow cytometric staining, the suggested use of this reagent is ≤0.25 µg per million cells in 100 µl volume. It is recommended that the reagent be titrated for optimal performance for each application.
11) FITC anti-mouse CD45 Antibody (clone: 30-F11)
Verified Reactivity: Mouse, Application: FC - Quality tested
Each lot of this antibody is quality control tested by immunofluorescent staining with flow cytometric analysis. For flow cytometric staining, the suggested use of this reagent is ≤ 0.25 µg per 106 cells in 100 µl volume. It is recommended that the reagent be titrated for optimal performance for each application.
12) Alexa Fluor 488 anti-mouse CD45.2 Antibody (clone: 104, catalog #: 109816)
Verified Reactivity: Mouse, Application: FC - Quality tested
Each lot of this antibody is quality control tested by immunofluorescent staining with flow cytometric analysis. For flow cytometric staining, the suggested use of this reagent is ≤ 0.25 µg per 106 cells in 100 µl volume. It is recommended that the reagent be titrated for optimal performance for each application.
13) PE anti-mouse CD34 Antibody (clone: SA376A4)
Verified Reactivity: Mouse, Application: FC - Quality tested
Each lot of this antibody is quality control tested by immunofluorescent staining with flow cytometric analysis. For flow cytometric staining, the suggested use of this reagent is ≤1.0 µg per million cells in 100 µl volume. It is recommended that the reagent be

titrated for optimal performance for each application.
14) PE anti-mouse CD63 Antibody (clone: NVG-2)
Verified Reactivity: Mouse, Application:ICFC - Quality tested, FC - Verified
Each lot of this antibody is quality control tested by intracellular immunofluorescent staining with flow cytometric analysis. For intracellular flow cytometric staining, the suggested use of this reagent is ≤ 0.5 µg per million cells in 100 µL volume. For flow cytometric staining, the suggested use of this reagent is ≤ 0.5 µg per million cells in 100 µL volume. It is recommended that the reagent be titrated for optimal performance for each application.
15) PE anti-mouse CD371 (CLEC12A) Antibody (clone: 5D3/CLEC12A, catalog #: 143404)
Verified Reactivity: Mouse, Application: FC - Quality tested
Each lot of this antibody is quality control tested by immunofluorescent staining with flow cytometric analysis. For flow cytometric staining, the suggested use of this reagent is ≤0.25 µg per million cells in 100 µl volume. It is recommended that the reagent be titrated for optimal performance for each application.
16) PE anti-mouse CD184 (CXCR4) Antibody (clone: L276F12, catalog #: 146506)
Verified Reactivity: Mouse, Application: FC - Quality tested
Each lot of this antibody is quality control tested by immunofluorescent staining with flow cytometric analysis. For flow cytometric staining, the suggested use of this reagent is ≤0.5 µg per million cells in 100 µl volume. It is recommended that the reagent be titrated for optimal performance for each application
17) PE Rat IgG2a, κ Isotype Ctrl Antibody (clone: RTK2758)
Host Species: Rat, Immunogen: Trinitrophenol + KLH, Application: FC - Quality tested, ICFC - Verified
Each lot of this antibody is quality control tested by immunofluorescent staining with flow cytometric analysis as negative control. Use at concentrations comparable to those of the specific antibody of interest.
18) PE Rat IgG2b, κ Isotype Ctrl Antibody (clone: RTK4530, catalog #: 400608)
Host Species: Rat, Immunogen: Trinitrophenol + KLH, Application: FC - Quality tested, ICFC - Verified
Each lot of this rat IgG2b, κ isotype control antibody is quality control tested by immunofluorescent staining with flow cytometric analysis. For flow cytometric staining, use the isotype control at the same concentration as your primary antibody. Use our Concentration Lookup tool to find the exact concentrations of your lots of product.
19) PE/Cyanine7 anti-mouse CD49b Antibody (clone: HMa2)
Verified Reactivity: Mouse, Application: FC - Quality tested
Each lot of this antibody is quality control tested by immunofluorescent staining with flow cytometric analysis. For flow cytometric staining, the suggested use of this reagent is ≤0.125 µg per million cells in 100 µl volume. It is recommended that the reagent be titrated for optimal performance for each application.
20) PE/Cyanine7 anti-mouse CD200R3 Antibody (clone: Ba13)
Verified Reactivity: Mouse, Application: FC - Quality tested
Each lot of this antibody is quality control tested by immunofluorescent staining with flow cytometric analysis. For flow cytometric staining, the suggested use of this reagent is = 0.25 µg per million cells in 100 µl volume. It is recommended that the reagent be titrated for optimal performance for each application.
21) APC anti-mouse CD200R3 Antibody (clone: Ba13)
Verified Reactivity: Mouse, Application: FC - Quality tested
Each lot of this antibody is quality control tested by immunofluorescent staining with flow cytometric analysis. For flow cytometric staining, the suggested use of this reagent is ≤0.25 µg per million cells in 100 µl volume. It is recommended that the reagent be titrated for optimal performance for each application.
22) APC anti-mouse Ly-6A/E (Sca-1) Antibody (clone: D7, catalog #: 108112)
Verified Reactivity: Mouse, Application: FC - Quality tested
Each lot of this antibody is quality control tested by immunofluorescent staining with flow cytometric analysis. For flow cytometric staining, the suggested use of this reagent is ≤0.25 µg per million cells in 100 µl volume. It is recommended that the reagent be titrated for optimal performance for each application.
23) APC anti-mouse CD371 (CLEC12A) Antibody (clone: 5D3/CLEC12A)
Verified Reactivity: Mouse, Application: FC - Quality tested
Each lot of this antibody is quality control tested by immunofluorescent staining with flow cytometric analysis. For flow cytometric staining, the suggested use of this reagent is ≤0.25 µg per million cells in 100 µl volume. It is recommended that the reagent be titrated for optimal performance for each application.
24) APC/Cyanine7 anti-mouse CD16/32 Antibody (clone: S17011E)
Verified Reactivity: Mouse, Application: FC - Quality tested
Each lot of this antibody is quality control tested by immunofluorescent staining with flow cytometric analysis. For flow cytometric staining, the suggested use of this reagent is = 0.25 µg per million cells in 100 µl volume. It is recommended that the reagent be titrated for optimal performance for each application.
25) APC/Fire 750 anti-mouse CD9 Antibody (clone: MZ3)
Verified Reactivity: Mouse, Application: FC - Quality tested
Each lot of this antibody is quality control tested by immunofluorescent staining with flow cytometric analysis. For flow cytometric staining, the suggested use of this reagent is ≤ 0.5 µg per million cells in 100 µl volume. It is recommended that the reagent be titrated for optimal performance for each application.
26) Pacific Blue anti-mouse CD117 (c-Kit) Antibody (clone: 2B8)
Verified Reactivity: Mouse, Application: FC - Quality tested
Each lot of this antibody is quality control tested by immunofluorescent staining with flow cytometric analysis. The suggested use of this reagent is ≤ 1.0 µg per 106 cells in 100 µl volume. It is highly recommended that the reagent be titrated for optimal performance for each application.
27) Pacific Blue anti-mouse CD45.1 Antibody (clone: A20)
Verified Reactivity: Mouse, Application: FC - Quality tested
Each lot of this antibody is quality control tested by immunofluorescent staining with flow cytometric analysis. The suggested use of this reagent is ≤ 1.0 µg per 106 cells in 100 µl volume. It is highly recommended that the reagent be titrated for optimal performance for each application.

28) Brilliant Violet 421 anti-mouse CD34 Antibody (clone: SA376A4, catalog #: 152208)
Verified Reactivity: Mouse, Application: FC - Quality tested
Each lot of this antibody is quality control tested by immunofluorescent staining with flow cytometric analysis. For flow cytometric staining, the suggested use of this reagent is ≤0.5 µg per million cells in 100 µl volume. It is recommended that the reagent be titrated for optimal performance for each application.
29) Brilliant Violet 421 anti-mouse CD45 Antibody (clone: 30-F11, catalog #: 103134)
Verified Reactivity: Mouse, Application: FC - Quality tested, SB - Reported in the literature, not verified in house
Each lot of this antibody is quality control tested by immunofluorescent staining with flow cytometric analysis. For immunofluorescent staining using the µg size, the suggested use of this reagent is ≤0.25 µg per million cells in 100 µl volume. For immunofluorescent staining using the µl size, the suggested use of this reagent is 5 µl per million cells in 100 µl staining volume or 5 µl per 100 µl of whole blood. It is recommended that the reagent be titrated for optimal performance for each application.
30) Brilliant Violet 510 anti-mouse FcεRIα Antibody (clone: MAR-1, catalog #: 134327)
Verified Reactivity: Mouse, Application: FC - Quality tested
Each lot of this antibody is quality control tested by immunofluorescent staining with flow cytometric analysis. For flow cytometric staining, the suggested use of this reagent is ≤ 0.5 µg per million cells in 100 µl volume. It is recommended that the reagent be titrated for optimal performance for each application.
31) Brilliant Violet 605 anti-mouse CD117 (c-Kit) Antibody (clone: 2B8, catalog #: 105847)
Verified Reactivity: Mouse, Application: FC - Quality tested
Each lot of this antibody is quality control tested by immunofluorescent staining with flow cytometric analysis. For flow cytometric staining, the suggested use of this reagent is ≤ 0.25 µg per million cells in 100 µl volume. It is recommended that the reagent be titrated for optimal performance for each application.
32) Brilliant Violet 711 anti-mouse CD184 (CXCR4) Antibody (clone: L276F12, catalog #: 146517)
Verified Reactivity: Mouse, Application: FC - Quality tested
Each lot of this antibody is quality control tested by immunofluorescent staining with flow cytometric analysis. For flow cytometric staining, the suggested use of this reagent is ≤1.0 µg per million cells in 100 µl volume. It is recommended that the reagent be titrated for optimal performance for each application.
33) Brilliant Violet 785 anti-mouse CD127 (IL-7Rα) Antibody (clone: A7R34, catalog #: 135037)
Verified Reactivity: Mouse, Application: FC - Quality tested
Each lot of this antibody is quality control tested by immunofluorescent staining with flow cytometric analysis. For flow cytometric staining, the suggested use of this reagent is ≤0.5 µg per million cells in 100 µl volume. It is recommended that the reagent be titrated for optimal performance for each application.
34) PerCP/Cyanine5.5 anti-mouse TER-119/Erythroid Cells Antibody (clone: TER-119, catalog #: 116228).
Verified Reactivity: Mouse, Application: FC - Quality tested
Each lot of this antibody is quality control tested by immunofluorescent staining with flow cytometric analysis. For flow cytometric staining, the suggested use of this reagent is ≤ 0.25 µg per 106 cells in 100 µl volume. It is recommended that the reagent be titrated for optimal performance for each application.
35) TruStain FcX™ PLUS (anti-mouse CD16/32) Antibody
Verified Reactivity: Mouse, Application: FC - Quality tested
For blocking of Fc receptors in flow cytometric analysis, pre-incubate the cells with TruStain FcX™ PLUS for 5-10 minutes, on ice, at 0.25 µg per 106 cells in a volume of 100 µl, prior to immunostaining. It is not necessary to wash the cells between the blocking and immunostaining steps.
36) BD OptiBuild BV711 Hamster Anti-Mouse CD49b (clone: HMa2)
Reactivity: Mouse (Tested in Development), Application: Flow cytometry (Qualified)
This antibody was developed for use in flow cytometry.
The production process underwent stringent testing and validation to assure that it generates a high-quality conjugate with consistent performance and specific binding activity. However, verification testing has not been performed on all conjugate lots.
Researchers should determine the optimal concentration of this reagent for their individual applications.
37) FITC-conjugated Streptavidin (catalog #: 11-4317-87) .
Reported Application: Flow Cytometric Analysis, Immunocytochemistry, Immunohistochemical Staining of Frozen Tissue Sections
38) LEAF Purified anti-mouse IL-3 Antibody
Verified Reactivity: Mouse, Application: ELISA Capture - Quality tested, ELISPOT Capture, Neut - Reported in the literature, not verified in house
Each lot of this antibody is quality control tested by ELISA assay. For ELISPOT applications, a concentration range of 2.0 - 8.0 µg/mL is recommended. For ELISA capture applications, a concentration range of 0.5 - 2.0 µg/mL is recommended. To obtain a linear standard curve, serial dilutions of IL-3 recombinant protein ranging from 250 to 2 pg/mL are recommended for each ELISA plate. It is recommended that the reagent be titrated for optimal performance for each application.
39) TotalSeq-anti-mouse Hashtag 1 Antibodies
Verified Reactivity: Mouse, Application: PG - Quality tested
Each lot of this antibody is quality control tested by immunofluorescent staining with flow cytometric analysis and the oligomer sequence is confirmed by sequencing. TotalSeq™-A antibodies are compatible with 10x Genomics Single Cell Gene Expression Solutions.
To maximize performance, it is strongly recommended that the reagent be titrated for each application, and that you centrifuge the antibody dilution before adding to the cells at 14,000xg at 2 - 8°C for 10 minutes. Carefully pipette out the liquid avoiding the bottom of the tube and add to the cell suspension. For Proteogenomics analysis, the suggested starting amount of this reagent for titration is ≤ 1.0 µg per million cells in 100 µL volume. Refer to the corresponding TotalSeq™ protocol for specific staining instructions.
40) Validation of TNP-specific IgE antibody (IgE-Lb4, purchased from ADCC) was conducted in our laboratory. 1 µg/mL of this antibody was sufficient to sensitize bone marrow-derived basophils.

Animals and other research organisms

Policy information about studies involving animals; ARRIVE guidelines recommended for reporting animal research, and Sex and Gender in Research

Laboratory animals	7-10 week-old mice were used in this study. C57BL/6J and BALB/c wild-type mice were purchased from Japan SLC. Mcpt8GFP transgenic mice were described in Miyake et al. PNAS 2017. IL-4 reporter G4 (Il4GFP/+) mice were described in Hu-li et al. Immunity 2001. CD45.1 congenic mice were purchased from the Jackson Laboratory. Mice were maintained under specific pathogen-free conditions in our animal facilities. Animal rooms are maintained at 22±3°C, with a 12h:12h light-dark cycle, and humidity is maintained between 30%-70%. Animals are housed in individually ventilated cages (Innovive Caging System) and are fed with natural ingredient chow diet ad libitum (Japan CLEA: Rodent Diet CE-2). Cages are bedded with cloth material (Japan SLC: Q-pura chip). Animals are group housed whenever possible.
Wild animals	No wild animals were used in this study.
Reporting on sex	Data presented here is based on female mice, but the findings presented in this study were not restricted to female mice.
Field-collected samples	No field collected samples were used in this study.
Ethics oversight	All animal studies in this study were approved by the Institutional Animal Care and Use Committee of Tokyo Medical and Dental University (approval No. A2022-023C).

Note that full information on the approval of the study protocol must also be provided in the manuscript.

Flow Cytometry

Plots

Confirm that:

- [x] The axis labels state the marker and fluorochrome used (e.g. CD4-FITC).
- [x] The axis scales are clearly visible. Include numbers along axes only for bottom left plot of group (a 'group' is an analysis of identical markers).
- [x] All plots are contour plots with outliers or pseudocolor plots.
- [x] A numerical value for number of cells or percentage (with statistics) is provided.

Methodology

Sample preparation	Single cell suspensions were prepared from the bone marrow and spleen, followed by red blood cell (RBC) lysis by using RBC Lysis Buffer (BioLegend) for 5 min. For the flow cytometric analysis of peripheral blood, leukocytes from the heparinized blood sample were isolated from the interphase of 25% and 65% Percoll PLUS (cytiva) after gradient centrifugation. For the flow cytometric analysis of lung and skin samples, tissues were minced by razors and dissociated by using gentleMACS dissociator (Miltenyi Biotec) and Lung Dissociation Kit (Miltenyi Biotec) or Multi Tissue Dissociation Kit 1 (Miltenyi Biotec). Cells were stained with the indicated antibodies after treatment with normal rat serum (Merck Millipore) and TrueStain FcX PLUS (2.5 ug/mL; BioLegend) to prevent non-specific binding.
Instrument	Flow-cytometric analysis was performed using BD FACS Lyric flow cytometer (BD Biosciences). Cell sorting was performed using BD FACS AriaIII (BD Biosciences).
Software	Flow cytometry data were analyzed using FlowJo software (version 10.8.1, BD Biosciences)
Cell population abundance	Purity for all cell populations were confirmed to be greater than 95%.
Gating strategy	Basophils were gated as CD200R3+cKit- cells or CD49b+CD200R3+cKit- cells. Pre-basophils and mature basophils were gated as CLEC12AhiCD9lo and CLEC12AloCD9hi cells among basophil population, respectively. BaPs were gated as Cd34 +CLEC12AhiCD9loCD200R3+cKit- cells. pre-BMPs were gated as Lin-FcεR1α+IL-7Rα-Sca-1-cKit+CD34+ cells.

- [x] Tick this box to confirm that a figure exemplifying the gating strategy is provided in the Supplementary Information.
